# Evaluation of the Effects of *Punica granatum* Flower Tablets on Pain and Healing of Minor Recurrent Aphthous Stomatitis: A Randomized Clinical Trial

**DOI:** 10.1002/cre2.920

**Published:** 2024-07-17

**Authors:** Fatemeh Lavaee, Elahehnaz Parhizkar, Fatemeh Tavakoli, Mohammad M. Zarshenas, Naeimehossadat Asmarian

**Affiliations:** ^1^ Oral and Maxillofacial Disease Department, School of Dentistry Shiraz University of Medical Sciences Shiraz Iran; ^2^ Department of Pharmaceutics, School of Pharmacy Shiraz University of Medical Sciences Shiraz Iran; ^3^ Oral and Maxillofacial Medicine Department, School of Dentistry Shiraz University of Medical Sciences Shiraz Iran; ^4^ Department of Phytopharmaceuticals (Traditional Pharmacy), School of Pharmacy Shiraz University of Medical Sciences Shiraz Iran; ^5^ Anesthesiology and Critical Care Research Center Shiraz University of Medical Sciences Shiraz Iran

**Keywords:** *Punica granatum* flower tablet, recurrent aphthous stomatitis, triadent

## Abstract

**Objectives:**

The purpose of this study was to determine the therapeutic effect of the *Punica granatum* (PG) flower on recurrent aphthous stomatitis in comparison with corticosteroid therapy.

**Materials and Methods:**

This cross‐over randomized clinical trial was conducted on the patients who had been referred to Shiraz Dental School for their RAS in 2021. All the participants used both *P. granatum* flower tablets and Triadent a month apart for wash‐out time and all compared themselves. In the experimental group, 30 patients received pomegranate flower tablets, three tablets daily, for 6 days. In the control group, oral paste Triadent has been prescribed three times a day for 6 days. The visual analog scale (VAS) and the size of RAS were evaluated on Days 0–6. Data were analyzed by SPSS version 21. The Wilcoxon test was used.

**Results:**

The mean age of participants was 27.8 ± 14.77 years old. In this study, 15 patients (50%) were men and 15 patients (50%) were women. The mean value of VAS after using prescribed treatment in both evaluated groups on all days was significantly different such that the VAS values were lower for PG flower tablets than Triadent (*p* value < 0.05). The size of oral lesions in participants who used PG flower tablets was significantly less than those who used Triadent on all evaluation days (*p* value < 0.05) except on Day 1 (*p* value = 0.29). The descending slope of VAS from Days 1 to 6 for both Triadent and PG flower tablet users was significant and noticeable. (*p* value < 0.05).

**Conclusion:**

According to the result of this study, both *P. granatum* flower tablet and Triadent are useful in reducing the size, period of healing, and VAS of patients with RAS, but the PG flower tablet is more effective.

## 1 Introduction

Recurrent aphthous stomatitis (RAS) is one of the most common problems recognized by both dentists and skin specialists (Ghalayani et al. 2013). Every year about 2% to 66% of people all around the world are infected (Tarakji et al. 2015). Aphthous is the most common oral lesion and most commonly occurs in young ages between 25 and 35 years old (Hasan et al. 2022). In addition, there is a direct association between the incidence of infection and age, gender, and socioeconomic status (Axéll and Henricsson 1985).

Aphthous ulcers occur on non‐keratinized mucosa, such as tongue edge, buccal mucosa, and lips (Yogarajah and Setterfield 2021). These ulcers are characterized by painful, recurring, small oral mucosal ulcers with a round or oval lining (Chavan et al. 2012). Aphthous ulcers are classified into three types: major, minor, and herpetic forms. The most common type is minor RAS, with an incidence of 70% and a lesion diameter much less than 1 cm (Hamedi et al. 2016; Elias, Fatahzadeh, and Schwartz 2022). A minor RAS has a clear margin and cures within 10–14 days. Minor RAS manifests on non‐keratinized mucosa, making it infrequent on the dorsum of the tongue, palate, or gingiva. Its prevalence is higher in the floor of the mouth, buccal mucosa, and labial mucosa. Lesions predominantly occur in the anterior region of the oral cavity and are characterized by superficial ulcers (Martin et al. 2008). In the major type, the prevalence is between 7% and 20%, the diameter of the lesion is greater than 1 cm, and dysphagia may take place (Queiroz et al. 2018). Treatment can last up to several weeks. Herpetic form rarely occurs and is associated with small multiple ulcers; healing may take 7–10 days (Ship 1996). This type of appearance is similar to herpetic lesions, but the herpes simplex virus is not found in culture (Burket, Greenberg, and Glick 2003). RAS is a multifactor process. Genetic predisposition, hematological anomalies, microbial or immunological factors, trauma, stress, and hormonal status are important predisposition factors for this condition (Sudheesh and Vijayalakshmi 2005; Borhani Haghighi and Bagheri 2007; Boras et al. 2006; Khademi et al. 2014; Eslami et al. 2023). Because of this multifactor etiology, there is no definitive treatment for RAS. The main aims of the therapy are palliative, the prevention of recurrence, and the promotion of cure of ulcers (Khademi et al. 2014; Rosenblat et al. 2006; Jurenka 2008). Topical corticosteroids, analgesics, and antibiotics are highly recommended for patients with RAS (Milia et al. 2022; Lau and Smith 2022) The best treatment for RAS is the topical application of medications (García‐Feijoo et al. 2015). Not only because the drug may have a direct influence on the source of the disease, but also because the drug's systemic side effects are minimized (Tavangar, Aslani, and Nikbakht 2019; Eslami et al. 2022). Natural medicinal plants as an alternative therapy for aphthous have been widely used in many countries for decades. Favorable advantages of such remedies have been shown by clinical studies. They are effective in reducing the discomfort and duration of ulcers (Braga et al. 2005; Chidambara Murthy, Jayaprakasha, and Singh 2002). *Punica granatum* (PG) has been introduced as a natural medicine for preventing and treating inflammation and cancer (Eltay et al. 2021). PG is a food complement that contains flavonoids. PG flavonoids showed antimicrobial activity, a capacity to sweep free radicals, activation of the immune system, and several antioxidant properties (Ghalayani et al. 2013). These characteristics of PG may be advantageous for the treatment of RAS (Salahvarzi, Tehranifar, and Jahanbakhsh 2011). *P. granatum* flowers (Golnar) have been used for mouth and anal ulcers, intranasal ulcers, wounds between the toes, peptic ulcers, and ear pain. In Iranian medicine, Golnar is said to have a reinforcing effect on the gums and may be useful for loose teeth. Its application to scrapes or wounds may quickly cure them (Gavanji, Larki, and Bakhtari 2014). Disruptions in the activity of antioxidants and the immune system are considered important factors in the aphthous etiology (Saraswathi Gopal et al. 2021). As this disease is accompanied by inflammation and pain, the pomegranate flower can be used to relieve symptoms and shorten the duration of the disease due to its antioxidant, antimicrobial, anti‐inflammatory, analgesic, and healing properties (Albahri et al. 2023; Gandhi et al. 2020) Furthermore, the present tannins creates a protecting layer by causative proteins thereby preventing the ulceration from turning into infection or worse (Kaur et al. 2006).

The pomegranate tree can be divided into multiple anatomic compartments: seeds, barks, juices, flowers, leaves, and roots. All of them have interesting pharmacologic activities. It has also been demonstrated that the use of juices, peel, and oil provides anti‐cancer activities, including interference with tumor cell proliferation, invasion, cell cycle, and angiogenesis. These effects could also be regarding plant‐derived anti‐inflammatory drug effects (Lansky and Newman 2007). The phytochemical and pharmacological actions of all PG ingredients suggest ointment, creams, pastes, emulsions, and gels are topical formulations for diseases like aphthous (Kulawik‐Pióro and Goździcka 2022). Patient compliance and acceptance are critically vital for oral topical products. Ointments, creams, and some emulsions are seldom used for oral topical treatment and are less well accepted for application in the mouth (Aslani, Zolfaghari, and Davoodvandi 2016). The base of the formulation should have acceptable mucoadhesion so that the drug stays on site for longer. The buccal mucosa is one such mucosal site that has a high level of vascularization and allows direct outflow of blood flow to the jugular vein. Buccal delivery, therefore, means that the drug is easily absorbed through the oral and buccal mucosa (Smart 2005; Madhav et al. 2009). In this study, we aim to assess the effect of the *P. granatum* flower tablet and examine whether it can reduce pain and healing of RAS or not.

## 2 Methods and Material

Persian Golnar with the scientific name of *P. granatum* var. Hayne pleniflora belongs to the Punicae family. Golnar Farsi is a subspecies of pomegranate and is a male pomegranate plant that has only flowers and does not produce fruit. Persian Golnar is a thorny shrub or a short tree without thorns. The leaves are 1–4 cm long and 2–7 cm wide, rectangular bayonets or ovate, without hairs and the flowers are 3–4 cm in diameter. The petals are 2–5 cm long and 2–3 cm wide, rarely white. The flowers are two rows of fiery red. This plant is distributed in Golestan, Gorgan, Mazandaran, Gilan, Kurdistan, Lorestan, and Sistan and Baluchestan provinces as well as in the Mediterranean regions of Europe, Africa, Asia, Anatolia, Afghanistan, and Pakistan.

### 2.1 Persian Golnar Plant Identification

According to the purpose of the research which was an examination of the reduction of Inflammation, among the various medicinal plants, the Persian Golnar plant has been selected; in addition to the possible effective effects mentioned above, it is very available and acceptable in terms of price. For this purpose, three different samples of Golnar Farsi were purchased from three centers for the sale of medicinal plants. After transfer to the Faculty of Pharmacy and review by the Master of Plant Systematics, a sample was selected from the purchased samples taking into account the organoleptic characteristics and after obtaining the identification number, it was placed on the agenda of extracting and preparing the product. The plant was kept in the Shiraz School of Pharmacy with the identification number 1282PM.

### 2.2 Preparation of Extract

In this study, the hydroalcoholic extraction method, due to the release and extraction of more active substances from the hydroalcoholic extract, was put on the agenda instead of water extraction. An 80% hydroalcoholic solution was used to prepare the extract (99.6% alcohol without a biting agent).

For this purpose, after preparing the above solution, 1 kg of the Persian Golnar plant was powdered in a Kenwood electric grinder and passed through a sieve with 40 mesh. For extraction, it enters the percolator device, then a layer of filter paper is placed on the desired powder, and after pressing on the filter paper, the desired hydro‐alcoholic solution is gently poured on the powder in the amount of 5 L. The mixture was kept for 72 h. After this period, the percolator valve was opened slightly to establish the flow of the extract in drops. The prepared extract was poured into an amber glass jar and kept in the refrigerator for the next process.

#### 2.2.1 Concentration and Drying Step

At this stage, the obtained hydroalcoholic extract is concentrated in a rotary evaporator under vacuum and at a temperature of 50°C; after pouring it into 50 cc falcons, it is transferred to the speed vacuum device and completely concentrated in the mentioned device.

The rest of the solvent, which is mainly the aqueous phase, was placed in the freezer to dry to obtain a very soft dry powder (the Falcons were placed at minus 20°C for 24 h before entering the machine to completely freeze the liquid inside). The particle size of the dried extract was 600 nm. The final extract is prepared for the formulation phase after weighing and determining the extraction efficiency.

### 2.3 Preparation of Tablets

The tablets are prepared by the direct compression method. The formulation includes 50 mg of dry extract as API and sorbitol and lactose as tablet excipients. Magnesium stearate is added as a lubricant before compression. The powder is compressed on a tablet press (AR400, ERWEKA, Germany).

### 2.4 Evaluation of Tablets

In vitro decomposition time: 10 tablets were placed individually in 10 mL of distilled water and the time to complete decomposition was recorded.

### 2.5 Variation in Weight, Hardness, and Brittleness

Twenty pellets were randomly selected to weigh each pellet using a digital balance (Sartorius, Germany) and the mean and standard deviation of the weights were calculated. The hardness of the tablet was assessed using an ERWEKA hardness tester (Germany). An average hardness of 10 pellets was obtained. To measure the friability of the pellets, 10 pellets were weighed and placed in a friability tester rotating at 25 rpm for 4 min.

### 2.6 Determination of Pill Taste by Volunteers

The optimized tablet formulation and purified, mask‐free extract have been tested by 10 healthy volunteers. Taste is rated on a scale of 1 to 4; 1 (*very bitter*), 2 (*bitter*), 3 (*fair*), and 4 (*pleasant*).

#### 2.6.1 Tablet Evaluation

The disintegration time of tablets is between 180 and 300 s. The average pellet weight is 252.8 ± 3.2 and the average hardness of 10 pellets is 9.1 ± 0.2 kg. The friability of the 20 pellets was found to be within acceptable limits (< 1%). All of these physical characteristics of the tablets are within the acceptable limits recommended by the pharmacopeia.

#### 2.6.2 In Vivo Taste Masking of Tablets

All volunteers reported that the formulation made from the pure extract was very bitter (score 1), whereas the optimized formulation of the tablets was pleasing to all (score 4).

### 2.7 Study Design

This cross‐over randomized clinical trial was co on the patients who had been referred to Shiraz University of Medical Science, Shiraz Dental School for their RAS in 2021.

The protocol of the clinical trial was conducted according to the ethical principles of Helsinki (version 2002). The study has been approved by the Shiraz University of Medical Science ethical committee by the ethical code of IR.SUMS.DENTAL.REC.1399.136. Also, this study has been registered in the Iranian Registry of Clinical Trials by code IRCT20120101008585N12.

### 2.8 Patient Selection

Patients with minor RAS for less than 24 h have been enrolled in this research. The participant should not consume any other medication for their problem during this evaluation. They should not have used any topical or oral corticosteroid or colchicine since 2 months ago. They should not have any related systemic diseases such as Crohn's disease, Behcet disease, Reiter syndrome, or other inflammatory diseases. Also, they should not use any analgesic mouthwash, oral pastes, or systemic analgesics 14 days before this evaluation. Pregnant women were excluded too. The participants, who met the inclusion and exclusion criteria, were enrolled in this evaluation if they signed the written consent form.

With a 95% confidence interval and power of 80%, the total sample size was considered 30.

One of the researchers assigned the therapeutic intervention and the other one assessed the outcome. The patient himself recorded the size, pain, and healing time of the lesions. In this study, the outcome assessor and the statistical analyzer were blind. To determine the sample size, SPSS software is used.

A total of 100 patients signed the consent form and they were given PG. Flowers tablets for their first therapeutic medication. Finally, only 50 participants completed the evaluation. They received pomegranate flower tablets, three tablets daily, for 6 days. For the next recurrence of minor RAS, only 30 patients from those 50 experienced minor RAS again. Then, these 30 participants in the next recurrence of RAS, which was at least 1 month after the first occurrence, were given Triadent oral paste as a second therapeutic medication.

Oral paste Triadent 0.1% (Rahapharma, Isfahan, Iran) has been prescribed three times a day for 6 days as well.

By inserting the PG flower tablet into the lesion, the tablet itself attaches to the oral ulcer for some minutes because of the wetness of the mucosa and dryness of the tablet, then it dissolves in salvia. The patient should hold this tablet until it is dissolved completely. The patient is not required to retain saliva in the mouth during this period. The researcher evaluated the pain degree by visual analog scale (VAS) before and after using tablets and oral paste at both times on Days 1, 2, 3, 4, 5, and 6. The size of a specific ulcer in each patient also has been measured on Days 1, 2, 3, 4, 5, and 6. The size of a specific ulcer in each patient has been measured by a graded tongue blade by the patient himself. Patients were educated in filling out the form of data gathering, measuring the size of the lesion, and VAS.

In this study, the patients were not blind because of two different prescribed treatments in each group.

### 2.9 Data Analysis

Data were analyzed using SPSS 21 and *p* values < 0.05 were considered statistically significant. In this study, variables were reported as the median and the IQR. Wilcoxon test was used for analysis.

## 3 Results

**Figure 1:**
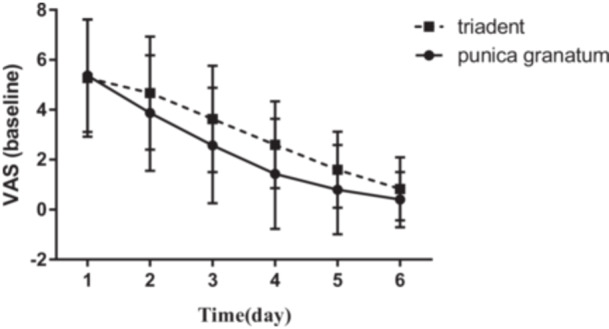
Comparison of baseline VAS between different days and between patients who used Triadent and PG tablets on each day.

**Figure 2:**
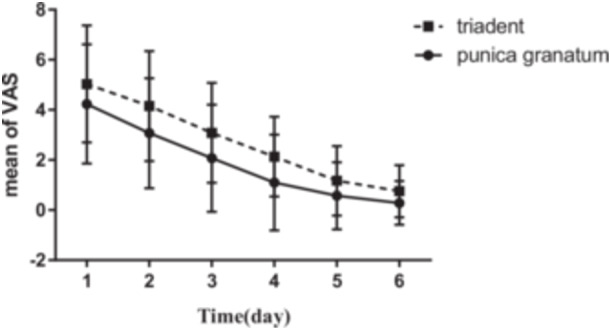
Comparison of VAS mean after using Triadent and PG tablets on different days and between patients who used Triadent and PG tablets on each day.

**Figure 3:**
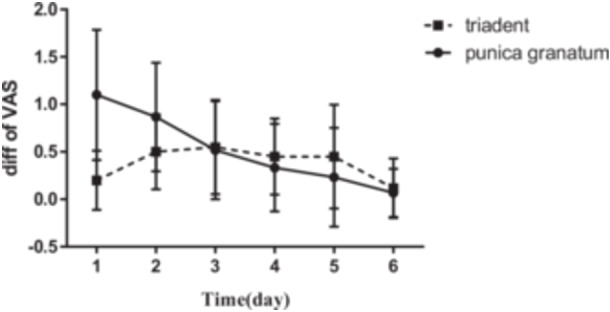
Comparison of VAS difference between different days and between patients who used Triadent and PG tablets on each day.

**Figure 4:**
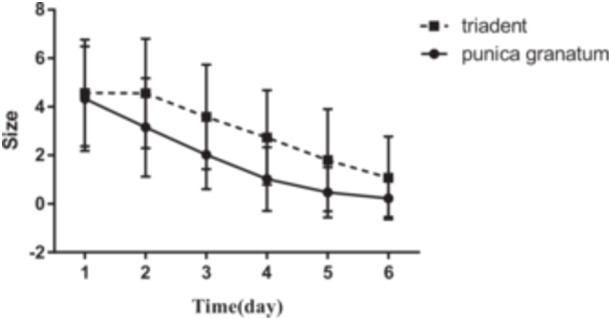
Comparison of the size of RAS lesions in millimeters between different days and between patients who used Triadent and PG tablets on each day.

The mean age of participants was 27.8 ± 14.77 years old. The mean values of VAS before and after using Triadent and PG flower tablets, and also the size of lesions on Days 1–6 are reported in Figures 1, 2, 3, 4.

In a comparison of two evaluated groups (Triadent vs. PG flower tablets), participants who had used PG flower tablets reported significantly lower VAS on all evaluation days (*p* value < 0.05) (before and after using treatment) except the first day (Figure 1). On the first day of evaluation, the VAS of recurrent aphthous lesions in both groups were not significantly different (*p* value = 0.74) before any intervention, which is valuable for a more homogenized and precise assessment.

The mean values of VAS after using prescribed treatment in both evaluated groups on all days were significantly different such that the VAS values were lower for PG flower tablets than Triadent (Figure 2).

The amount of VAS reduction on each day is compared between the two groups. The greatest reduction was on Days 1 and 2 when the RAS lesions had the largest size (Figure 3).

In addition, the size of oral lesions in participants who used PG flower tablets was significantly less than those who used Triadent on all evaluation days (*p* value < 0.05) except Day 1 (*p* value = 0.29) (Figure 4).

The median period of RAS healing in participants who used PG flower tablets was 3.5 days which was noticeably shorter than the 5 days for oral lesions in participants who used Triadent (*p* value = 0.001).

The mean of VAS values on each day has been compared to other days in each group. The descending slope of VAS from Days 1 to 6 for both Triadent and PG flower tablet users was significant and noticeable except on Day 3 in comparison to Day 4, and Day 4 in comparison to Day 5 for both groups. The amount of VAS decrease between some days in patients who had used Triadent was not substantial. (Table 1).

**Table 1:** Comparison of the VAS of RAS lesions between different days in each group.

Variable	*Punica granatum p* value	Triadent *p* value
VAS Day 1 vs. Day 2	0.16	0.004
VAS Day 1 vs. Day 3	0.002	0.005
VAS Day 1 vs. Day 4	0.001	0.004
VAS Day 1 vs. Day 5	< 0.0001	0.021
VAS Day 1 vs. Day 6	< 0.0001	0.454
VAS Day 2 vs. Day 3	0.007	0.72
VAS Day 2 vs. Day 4	< 0.0001	0.58
VAS Day 2 vs. Day 5	< 0.0001	0.75
VAS Day 2 vs. Day 6	< 0.0001	0.001
VAS Day 3 vs. Day 4	0.08	0.42
VAS Day 3 vs. Day 5	0.021	0.4
VAS Day 3 vs. Day 6	0.001	0.002
VAS Day 4 vs. Day 5	0.27	1.00
VAS Day 4 vs. Day 6	0.017	0.001
VAS Day 5 vs. Day 6	0.039	0.008

## 4 Discussion

The VAS and the size of lesions in participants who had been affected by RAS and had used PG flower tablets on all evaluated days were significantly lower than others who had used Triadent. In addition, the participants who had used PG flower tablets experienced a shorter period for RAS than the other group. The greatest decrease of VAS has been reported for Days 1 and 2 for PG flower tablets and this decrement was significant compared to Triadent. This finding confirms that the best effect of PG flower tablets was on the first 2 days of the RAS period.

Both prescribed treatments, PG flower tablets and Triadent decreased the size, pain, and period of RAS, but PG flower tablets were more successful. The amount of VAS reduction on each day is compared between the two groups. The largest VAS reduction was on Days 1 and 2 when the RAS lesions had the largest size. PG flower tablets showed a continuous alleviating effect on pain (VAS) and RAS healing. Its therapeutic effect began and continued as long as taking the PG flower tablets. While Triadent effects reached to optimum level during the first 2 days. The self‐limited nature of RAS can be demonstrated by approaching the mean VAS values in both groups in the last days of evaluation. Considering the amount of VAS reduction (VAS diff), it can be proposed that Triadent decreased the VAS more prominently in the first 3 days, while the effect of Triadent decreased in the remaining days. On the other hand, VAS reduction of PG flower tablets increased during the treatment period as long as taking the tablets. Ghalayani et al. (2013) designed a randomized double‐blind study. The participants used *P. granatum* gel or placebo gel randomly. Healing time and pain degree were recorded by each patient before (Day 0) and after (Days 1, 3, 5, and 7) using both gels. The findings showed *P. granatum* gel had a positive effect on reducing RAS pain and period of healing (*p* value < 0.001). This study was continued by Tavangar et al. Tavangar, Aslani, and Nikbakht (2019) evaluated the effect of *P. granatum* gel in comparison with Triadent. In their study, the newly designed *P. granatum* mucoadhesive gel had more effects on the duration of pain and time of healing in comparison with Triadent. These studies did not compare tissue repair and pain reduction on different days. Since RAS has a self‐limited pattern of healing, a comparison of different days can help to determine the therapeutic effect of *P. granatum* flower more precisely. In the presenting evaluation, the *P. granatum* flower was designed as a tablet that can adhere to oral mucosa for some minutes. All the participants used both *P. granatum* tablets and Triadent at least a month apart and this makes the comparison more precious since the personal factors have been controlled. In overall consideration, Triadent and *P. granatum* tablets are both effective on RAS treatment, while PG flower tablets had a more continuous alleviating effect on pain and RAS healing. RAS is a common, self‐limiting, inflammatory, painful disease in the oral cavity with an unknown etiology. It is identified by small ulcers with red margins (Rad et al. 2010) which has no specific treatment. Several factors contributed to RAS such as genetic pre‐disposition, hormonal state, stress, trauma, and microbial and or immunologic factors (Burket, Greenberg, and Glick 2003; Khademi et al. 2014). There are some suggestions for RAS treatment in traditional medicine, such as *P. granatum*'s different products. *P. granatum* is known as an anti‐inflammatory product that has been prescribed for oral lesions. These anti‐inflammatory properties are because of their polyphenolic compounds such as anthocyanins which can decrease IL‐2 and interferon‐gamma and TNF‐α. IL‐2 and interferon‐gamma are the main pathogenesis of RAS (Sudheesh and Vijayalakshmi 2005; Borhani Haghighi and Bagheri 2007; Boras et al. 2006). Also, it has been proven that *P. granatum* can remove free radicals and it can decrease oxidative stress and lipid peroxidation since animal studies have proven this property (Rosenblat et al. 2006; Jurenka 2008). All parts of *P. granatum* have anti‐inflammatory and antioxidative effects such as seed oil, juice, peel, and pulp (Jurenka 2008; Chidambara Murthy, Jayaprakasha, and Singh 2002). On the other hand, phenolic compounds of *P. granatum* showed antimicrobial effects (Güner and Aşkun 2023). A higher level of phenolics can induce more antioxidant and antimicrobial activity (Salahvarzi, Tehranifar, and Jahanbakhsh 2011). Gavanji, Larki, and Bakhtari (2014) compared the alcoholic and water extracts of *P. granatum* var. pleniflora, *P. granatum* var. Sweet Alak, and *P. granatum* var. Saveh Black on minor aphthous. The water and alcoholic extract of *P. granatum* var. pleniflora decreased the time of healing more than others due to a higher amount of phenolic compounds, resulting in higher antioxidant activity. *P. granatum* flower has a variety of secondary metabolites such as polyphenols with strong antioxidative activity (Ökmen et al. 2023). The polyphenols in *P. granatum* flowers contain alginic acid. The antibiotic activity of Persian golnar extract is due to its chemical components such as tannins and alkaloids found in its leaves, roots, stems, and fruits (Kaur et al. 2006). Pomegranate, which is widely cultivated in Iran, originates from the Middle East and Iran and has been used throughout the world over the years. Its widespread use is due to recent studies which show that this fruit contains high amounts of antioxidants that are beneficial to health. Its excellent taste and health benefits make it suitable for people who are looking for natural foods. Pomegranate is an important source of bioactive compounds and its various parts have been used in medicine for centuries. Since Triadent has been introduced as an effective treatment; in this study, the therapeutic effect of *P. granatum* flower tablets has been compared with Triadent. Triadent topical application as a corticosteroid can cause candidiasis, mucosal atrophy, burning mouth, hypogeusia, and inhibition of the hypothalamic‐pituitary‐adrenal axis in long‐term topical usage (George and Balan 2018). According to what this study revealed, *P. granatum* flower tablets were more effective than Triadent. Since it is an herbal product with fewer effects and more general acceptability in a different population, it can be prescribed instead of Triadent for RAS. On the other hand, RAS has a wet nature and according to traditional medicine, its treatment should have an opposite nature, that is, dry nature (Ge et al. 2021). *P. granatum* flower powder in the form of tablets can even prepare a dry nature for RAS treatment and also it can affect the lesion itself topically and for a longer duration in comparison to mouthwashes and gels. Some participants reported an acrid taste for these tablets and also a 4–5 s burning sensation. In our evaluation, we investigated the same patients in both evaluation and control groups. So the personal factors in pain perceptions and tissue healing have been homogenized. The effect of frequent *P. granatum* flower tablets usage for RAS may reduce the frequency of these lesions in a specific period. This can be assessed in future studies.

In previous studies, *P. granatum* extracts had been used in liquid or gel form which had been water‐based, while in the presenting study, these tablets are prepared in dry form for RAS treatment which is favorable on the basis of traditional medicine principles. Since RAS has a wet nature, designing a treatment considering the nature of the lesion (a tablet) can make the treatment more effective. This is the novelty of this study.

Limited sample size and longtime patient follow‐up are points which should be considered for future evaluations. Long time follow‐up can determine the preventive effect of using *P. granatum* flower tablets on RAS recurrence. Since oral mucosa in some locations are very mobile, the texture of this tablet for some locations in oral mucosa may be uncomfortable.

## 5 Conclusion

The VAS and the size of lesions in participants who had been affected by RAS and had used *P. granatum* flower tablets on all evaluated days were significantly lower than others who had used Triadent. In addition, participants who had used *P. granatum* flower tablets experienced a shorter period for RAS than the other group. *P. granatum* flower tablets showed a continuous alleviating effect on pain (VAS) and RAS healing. Its therapeutic effect began and continued as long as taking the *P. granatum* flower tablets. In contrast, Triadent effects reached to optimum level during the first 2 days.

## Author Contributions

E.P., F.L., and M.M.Z. developed the original idea and the protocol. N.A. analyzed data. F.T. wrote the manuscript and is a guarantor. F.T. abstracted data, and prepared the manuscript.

## Ethics Statement

The patients gave written informed consent, and the study was approved by the ethics committee of Shiraz University of Medical Sciences (IR.SUMS.DENTAL.REC.1399.136).

## Conflict of Interest

The authors declare no conflicts of interest.

## Data Availability

All data generated or analyzed during this study are included in this article.
